# A Three-Generation Family with Idiopathic Facial Palsy Suggesting an Autosomal Dominant Inheritance with High Penetrance

**DOI:** 10.1155/2015/683938

**Published:** 2015-01-18

**Authors:** Christian Grønhøj Larsen, Mette Gyldenløve, Aia Elise Jønch, Birgitte Charabi, Zeynep Tümer

**Affiliations:** ^1^Department of Otorhinolaryngology, Head and Neck Surgery, Copenhagen University Hospital of Rigshospitalet, 2100 Copenhagen, Denmark; ^2^Department of Dermato-Allergology, Gentofte Hospital, University of Copenhagen, 2900 Hellerup, Denmark; ^3^Clinical Genetic Clinic, Kennedy Center, Copenhagen University Hospital, Rigshospitalet, 2600 Glostrup, Denmark; ^4^Applied Human Molecular Genetics, Kennedy Center, Copenhagen University Hospital, Rigshospitalet, 2600 Glostrup, Denmark

## Abstract

Idiopathic facial palsy (IFP), also known as Bell's palsy, is a common neurologic disorder, but recurrent and familial forms are rare. This case series presents a three-generation family with idiopathic facial palsy. The mode of inheritance of IFP has previously been suggested as autosomal dominant with low or variable penetrance, but the present family indicates an autosomal dominant trait with high or complete penetrance. Chromosome microarray studies did not reveal a pathogenic copy number variation, which could enable identification of a candidate gene.

## 1. Introduction

Idiopathic facial palsy (IFP) (OMIM %134200), also known as Bell's palsy, is the sudden onset of paresis or paralysis of the seventh cranial nerve. It is a common neurologic disorder with an annual incidence of 13–52 cases per 100,000 individuals [1]. Typically, the patient presents with acute unilateral weakness of the facial muscles, alteration in taste, or both. In the majority of cases, full facial nerve function is reestablished [2], and partial recovery occurs within three weeks [2]. Although first described in 1821 by the Scottish surgeon Sir Charles Bell, the etiology of IFP remains largely unknown [3].

Most IFPs are sporadic, but both recurrent and familial forms have been described. Individuals with a single IFP are reported to have an 8% increased risk of recurrence [4, 5], and the risk is increased in families with multiples episodes of IFPs [6]. Overall, 2.4–28.6% of the IFPs are genetic [1]. Inherited, anatomical abnormality of the facial canal is the most common cause of the familial IFP [7], although several other causes have been associated with IFP, for example, vascular disease [8], immunogenic factors [9], and neurological disorders, such as the Melkersson-Rosenthal syndrome, Moebius syndrome, Charcot-Marie-Tooth disease, and hereditary neuropathies [1].

Previous case reports, where the most recent report is from 1990, suggest that the inheritance of familial IFP is autosomal dominant with low penetrance [8, 10, 11]. We here describe a three-generation family with six affected individuals where IFP segregates in an autosomal dominant manner with high or complete penetrance.

## 2. Clinical Report

**Figure 1:**
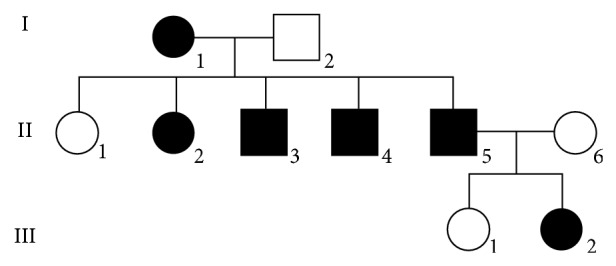
Family tree.

There are six affected individuals in the present family (Figure 1). Only the index patient (III: 2) has been clinically examined by the authors; information about the five other affected family members was obtained through III: 2 and II: 5.

### 2.1. Index Patient (III: 2)

A 26-year-old otherwise healthy woman presented with acute onset of left-sided facial muscle weakness and decreased left-sided taste sensation. She had no other symptoms or relevant expositions. According to the medical files, the patient had experienced two former IFPs occurring four months and five years previously. Both conditions had resolved completely within few months. The first IFP developed following a simple bacterial ear infection and the second IFP advanced without prelude of any kind. During childhood the patient suffered several middle ear infections.

On clinical examination the patient showed bilateral myringosclerosis and bilateral facial muscle weakness, most pronounced on the left side. In addition, both stapedial reflexes responded to high frequency tones only. Audiometric testing was normal, and magnetic resonance imaging of the cerebrum and the facial nerves revealed no abnormalities. Biochemical analyses were all normal, and Lyme disease due to a* Borrelia burgdorferi* infection was excluded by cerebrospinal fluid analysis. The patient was treated with oral prednisolone (2 × 25 mg/day) and acyclovir (5 × 800 mg/day) for 7 days. After two weeks, the patient had almost fully recovered with only minor muscle weakness remaining, which was still present two months later. Chromosome microarray analysis was carried out using CytoScan HD microarray platform (Affymetrix, USA) but did not reveal a microdeletion or duplication segregating with the disease in the family.

### 2.2. Other Family Members

The father (II: 5) of the index patient suffered a left-sided facial palsy when he was 25 years old. He consulted a physician, but no elucidation was performed and he did not receive any treatment. Today at 69 years of age, his face is still slightly asymmetric and the motor function of the lips is impaired.

Two paternal uncles (II: 4 and II: 3) of the index patient had a single IFP and were prescribed a corticosteroid injection and muscle-relaxing tablets, respectively. In both patients, the palsy disappeared completely within a month. The paternal aunt (II: 2) and grandmother (I: 1) both had an IFP incident during adolescence. They received no treatment or examination and showed complete remission.

## 3. Discussion

This case series presents a three-generation family, in which six individuals were affected with facial palsy. Except for a single episode following a bacterial ear infection, no triggering cause was identified in any of the patients.

There are only few reports of IFPs in the literature. To date, three families with three, four, and six affected individuals, respectively, have been described [1, 10, 11]. Based on inheritance patterns, we propose autosomal dominant inheritances with low or variable penetrance. This is supported by an epidemiological study from 1988, where Yanagihara et al. assessed 625 patients with IFP and found a positive family history in 26 of the patients [8]. The etiology of IFP is unclear, but the present case series supports the notion that genetic predisposition may play an important role. Patients with familial or recurrent IFPs require thorough clinical and paraclinical examination, and anatomical, vascular, immunological, and neurological disorders should be excluded as underlying factors.

Using high-resolution chromosome microarray analysis we could not detect a candidate susceptibility locus for IFP and further studies with whole genome exome sequencing of the current or other IFP families may lead to discovery of genes associated with familiar IFP.
